# Effect of physical activity for reducing anxiety symptoms in older adults: a meta-analysis of randomized controlled trials

**DOI:** 10.1186/s13102-024-00947-w

**Published:** 2024-07-16

**Authors:** Saba Goodarzi, Mohammad Mobin Teymouri Athar, Maryam Beiky, Hanieh Fathi, Zahra Nakhaee, Samira Parvizi Omran, Arman Shafiee

**Affiliations:** 1 Non-Communicable Diseases Research Center, Alborz University of Medical Sciences, Hassan Abad, Karaj, Alborz Province, Iran; 2 Student Research Committee, School of Medicine, Alborz University of Medical Sciences, Hassan Abad, Karaj, Alborz Province, Iran; 3 School of Medicine, Shahid Beheshti University of Medical Sciences, Tehran, Iran; 4 School of Medicine, Gonabad University of Medical Sciences, Gonabad, Iran; 5 School of Medicine, Tehran University of Medical Sciences, Tehran, Iran

**Keywords:** Physical activity, Exercise, Anxiety, Older adults

## Abstract

**Background:**

Anxiety symptoms in older adults can significantly impact their well-being. Physical activity is increasingly recognized as a potential intervention to alleviate anxiety in this population. We conducted a systematic review and meta-analysis to explore the impact of physical activity on anxiety symptoms in geriatric individuals.

**Methods:**

A systematic search was conducted in MEDLINE (via PubMed), Scopus, and Embase databases until November 29, 2023. Two independent reviewers screened articles based on predefined inclusion criteria.

**Results:**

Eleven randomized controlled trials were included. These trials, involving 770 geriatric participants, demonstrated a significant overall effect of physical activity on reducing anxiety symptoms (SMD =-0.60, 95% CI: -0.88 to -0.32). Subgroup analysis based on type of intervention and duration of follow-up was performed. The results showed all types of exercises reduced anxiety symptoms compared to the control group. Furthermore, those studies with shorter follow-up (less than 10 weeks) did not show a statistically significant reduction in anxiety symptoms. Moderate heterogeneity was observed (I2 = 67%). Sensitivity analyses confirmed the robustness of the overall effect size. Funnel plot inspection and Egger’s test (*p* = 0.36) suggested no signs of publication bias or small study effects.

**Conclusion:**

This meta-analysis provides strong evidence that physical activity significantly reduces anxiety symptoms in older adults. The study highlights the differential effects of cardio and strength exercises and underscores the high quality of evidence supporting the anxiolytic benefits of physical activity in geriatric populations.

## Introduction

Anxiety disorders are one the most prevalent category of mental health conditions and typically appear either before or during early adulthood [1]. Generalized anxiety disorder, social anxiety disorder, and panic disorder (with or without agoraphobia) are the most frequent anxiety disorders among the population [2]. They could also be accompanied by physical symptoms, such as shortness of breath, palpitations, and dizziness [2]. Of note, The World Health Organization (WHO) has classified anxiety disorders as the ninth most significant contributor to health-related disability, due to their high prevalence and morbidity [1, 3].

The process of aging is often associated with various physiological, psychological, and social challenges, potentially resulting in elevated levels of anxiety in the older population [4, 5]. The prevalence of anxiety in the older population is evaluated up to 15% in community samples [6]. Moreover, the impact of anxiety on the aging population could be significant and disabling [7]. Importantly, along with the aging of the global population, the prevalence of anxiety in older adults has turned out to be a serious public health concern, underscoring the necessity for effective interventions [8].

Various methods have been investigated and introduced to mitigate anxiety among this population, which are categorized into pharmacological [9] and non-pharmacological interventions, such as cognitive behavioral therapy and relaxation interventions [10–12]. For instance, music therapy has proven to be effective in managing anxiety in this population [13, 14]. Notably, a comparative analysis suggests the association of a sedentary lifestyle with higher levels of anxiety in the aging population, emphasizing the importance of physical activity in managing anxiety among the geriatric population [8]. Of note, various trials have evaluated the effect of physical activity in different forms such as pilates training, walking exercises, aerobic exercises, and resistance training, in managing anxiety in older adults [15–19]. The importance of aggregating the results of previous studies as a meta-analysis is evidenced in the literature [20]. Previous meta-analyses are available on this topic [21, 22]. Kazemnia et al. reported a decrease in anxiety symptoms after the sport intervention. Their study reported a comprehensive analysis of clinical trials; however, their conclusions were based on a before/after analysis [21, 23]. Before/after meta-analyses should be avoided for drawing evidenced based conclusions [23]. Another recent meta-analysis by Ofuso et al. [22] provided the results of 5 RCTs, however, additional RCTs are available on this topic. Hence, this systematic review and meta-analysis aims to review the published randomized clinical trial (RCT) studies addressing the effect of physical activity on anxiety among the geriatric population comparing it to the control group.

## Method

The present systematic review was conducted in accordance with the Cochrane Handbook and Preferred Reporting Items for Systematic Reviews and Meta-Analyses (PRISMA) guidelines [24, 25]. Our study protocol was retrospectively registered at PROSPERO under the number CRD42023488026.

### Search strategy

A systematic search was performed through MEDLINE (via PubMed), Scopus, and Embase databases until December 30, 2023. The following keywords were utilized as search strategy: (“physical activity” OR “exercise” OR “train*”) AND (“older adults” OR “elderly” OR “geriatric*”) AND (“anxiety”). No language restrictions were imposed.

### Study selection and data extraction

Two reviewers independently performed screening of the retrieved articles to include studies that met following inclusion criteria: (1) patient population: older adults (Above 60 years); (2) intervention: any cardio or resistance exercise that results in increase of heart rate; (3) comparison: active control groups which have not received any special recommendation for improving their physical activity; (4) outcome: anxiety level assessed by validated questionnaires; and (5) study design: only randomized controlled trial. Two reviewers will independently extract data from the included studies using a standardized data extraction form. The extracted information will include study characteristics (authors, year of publication, location), participant demographics, details of the physical activity intervention (type, duration, frequency), comparison group details, outcome measures, follow-up duration and main results. Any discrepancies between reviewers will be resolved through discussion or by consulting a third reviewer.

### Risk of bias assessment

The risk of bias of the included studies was assessed using the Cochrane Collaboration’s tool for assessing the risk of bias in randomized trials (RoB2). This tool evaluates potential bias in several domains, including random sequence generation, allocation concealment, blinding of participants and personnel, blinding of outcome assessment, incomplete outcome data, selective reporting, and other biases. Disagreements were solved by the third reviewer.

### Quality of evidence

The quality of evidence has been evaluated using the GRADE approach, and the results have been incorporated into the results section. The GRADE (Grading of Recommendations, Assessment, Development, and Evaluation) approach is a systematic method used to assess the quality of evidence and the strength of recommendations in healthcare. It categorizes evidence quality into four levels: high, moderate, low, and very low, considering factors such as study design, risk of bias, inconsistency, indirectness, imprecision, and publication bias. GRADE helps create evidence profiles and summary tables to present the findings clearly. This method ensures that clinical practice guidelines and systematic reviews are based on reliable evidence, ultimately guiding healthcare professionals in making well-informed decisions that balance benefits and risks for patients

**Fig. 1:**
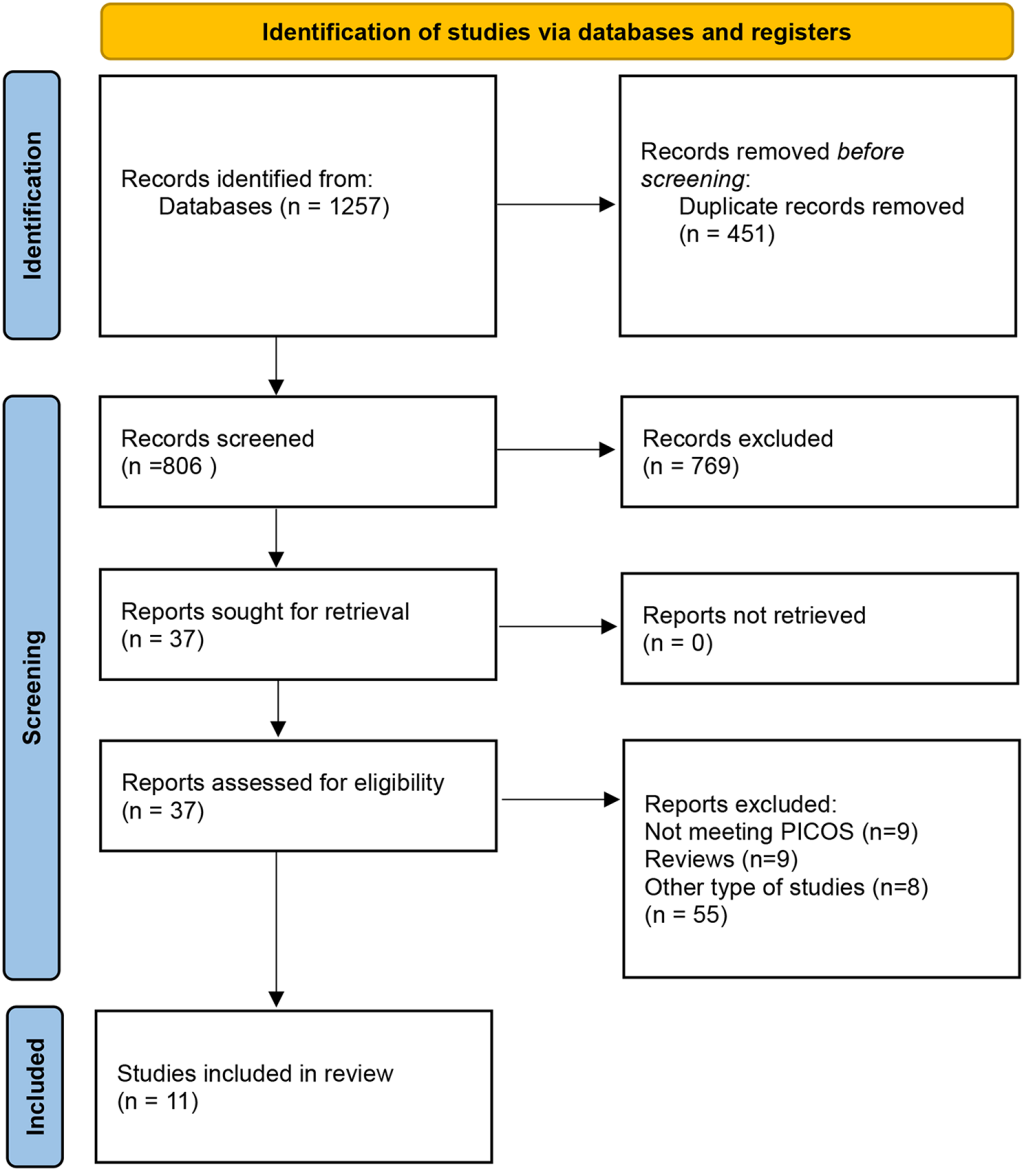
PRISMA flowchart

### Statistical analysis

To pool the effect sizes of the included studies, standardized mean differences (SMD) with their 95% confidence intervals (CI) were calculated using the post-intervention mean (SD) scores in both groups. A random effects model was employed to account for the potential heterogeneity among studies. The method used to estimate the between-study variance was the Restricted maximum likelihood (REML). Cochran’s Q statistic (Q-test) and the I² statistic were used to assess heterogeneity, with high heterogeneity defined as an I² value greater than 75%. For assessing possible publication bias, funnel plots and Egger’s regression test (with a significance level of 0.1) were used, provided there were at least 10 studies included. To perform sensitivity analysis, the leave-one-out method was applied to our outcome. Subgroup analyses were conducted based on the type of exercise and duration of follow-up. The analyses were considered significant with a p-value < 0.05. The R meta-package was utilized for all statistical analyses. The code used is publicly available on GitHub (https://github.com/Dr-Tlou/Code-for-meta-analysis).

## Results

### Study selection

The initial database search yielded a total of 1257 articles. After screening titles and abstracts, 37 articles were selected for full-text review. Ultimately, 11 randomized controlled trials (RCTs) met the inclusion criteria and were included in the meta-analysis [15, 17–19, 26–33] (Fig. 1).

### Characteristics of included studies

The included RCTs were conducted across diverse geographic locations and involved a total of 770 geriatric participants. The sample sizes of individual studies ranged from 14 to 203. The trials varied in duration, with intervention periods ranging from 6 weeks to 12 months. Interventions predominantly focused on various forms of physical activity, including aerobic exercises, resistance training, and combination programs. Baseline characteristics of the included studies is available in Table 1.

**Table 1:** Characteristics of the included studies

Author	Year of Publication	Country	Population	Sample Size (*n*)	Intervention	Control	Anxiety assessment	Follow up assessments
A. Aibar-Almazán	2019	Spain	Spanish postmenopausal women	107 Control (*n* = 52) Pilates (*n* = 55)	Pilates training		Hospital Anxiety and Depression Scale (HADS)	12 weeks
E. C. Chin	2022	Hong Kong	Older adults with insomnia	46	Walking sessions	stretching exercises	HADS	12 weeks
P. Deka	2021	USA	Patients with a diagnosis of CAD	90	high-intensity treadmill walking, resistance training		HADS	8 weeks
R. M. Ferreira	2018	USA	Individuals from the Parkinson’s Association in the State of Pará	35	resistance training		BAI	24 weeks
A. A. Ibrahim	2023	Saudi Arabia	Post-COVID-19 patients	72	walking on treadmill	Received medical care and advice	HADS	10 weeks
I. Imayama	2011	USA	Overweight/obese postmenopausal women	TOTAL = 439	aerobic exercise	not given an intervention	BSI-18	12 months
M. K. Mikkelsen	2022	Denmark	Older Patients with Advanced Cancer	84	Supervised exercise training: Warm up /PRT/ exercises / Relaxation Unsupervised exercise: Walking program	Step counting	HADS	13 weeks
A. Ruiz-Comellas	2022	Spain	Elderly with Anxiety, Depression, and Low Social Support	90	Walking	Usual care	GAD-7	4 months
R. W. S. Sit	2021	Hong Kong	Older Patients with chronic musculoskeletal pain	72	warming up (10 min), NM exercise (45 min), and cooling down (5 min).	Waiting-list	GAD-7	6 weeks
D. J. Yu	2022	Hong Kong	Middle-aged and older adults with mild cognitive impairment	37	walking group	stretching exercise	HADS	12 weeks
D. J. Yu	2023	Hong Kong	Middle-aged and older adults	30	walking exercise group	No intervention	GAD-7	12 weeks

### Effect of physical activity on anxiety symptoms

**Fig. 2:**
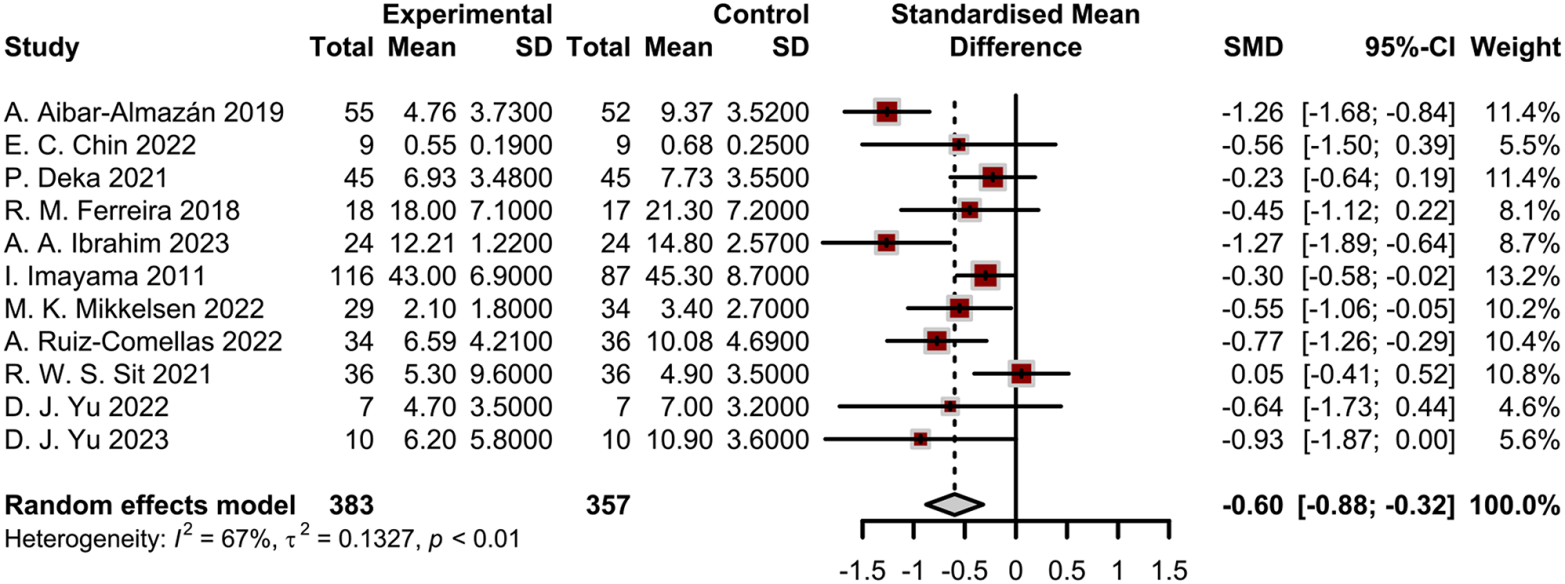
Results of meta-analysis

The meta-analysis revealed a significant overall effect of physical activity on reducing anxiety symptoms among geriatrics (SMD =-0.60, 95% CI: -0.88 to -0.32) (Fig. 2).

### Subgroup analysis

**Fig. 3:**
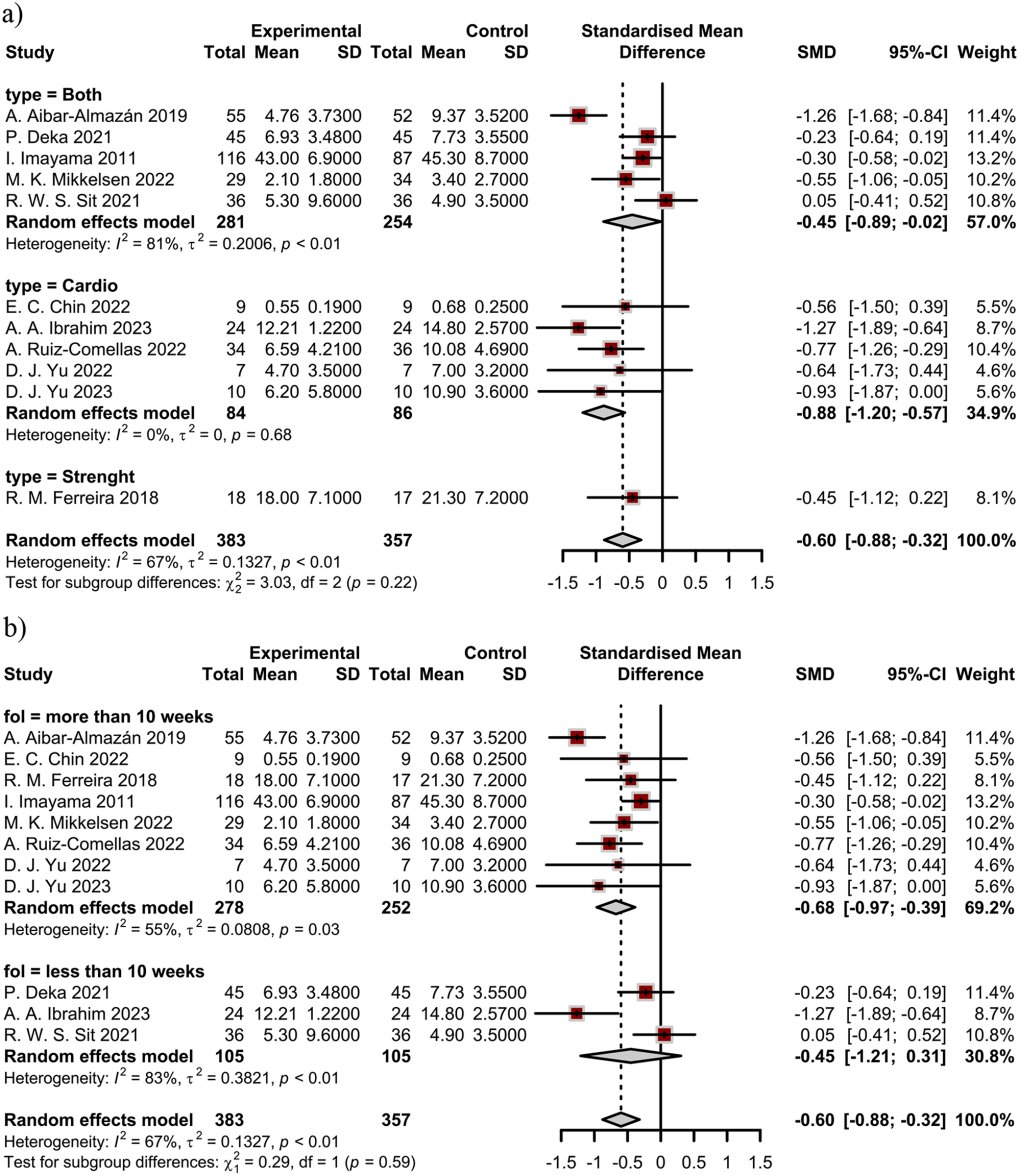
Results of subgroup analysis: (**a**) type of intervention; and (**b**) duration of follow-up

Subgroup analysis based on type of intervention and duration of follow-up was performed. The results showed all types of exercises reduced anxiety symptoms compared to the control group (Fig. 3). Furthermore, those studies with shorter follow-up (less than 10 weeks) did not show a statistically significant reduction in anxiety symptoms.

### Heterogeneity and sensitivity analysis

**Fig. 4:**
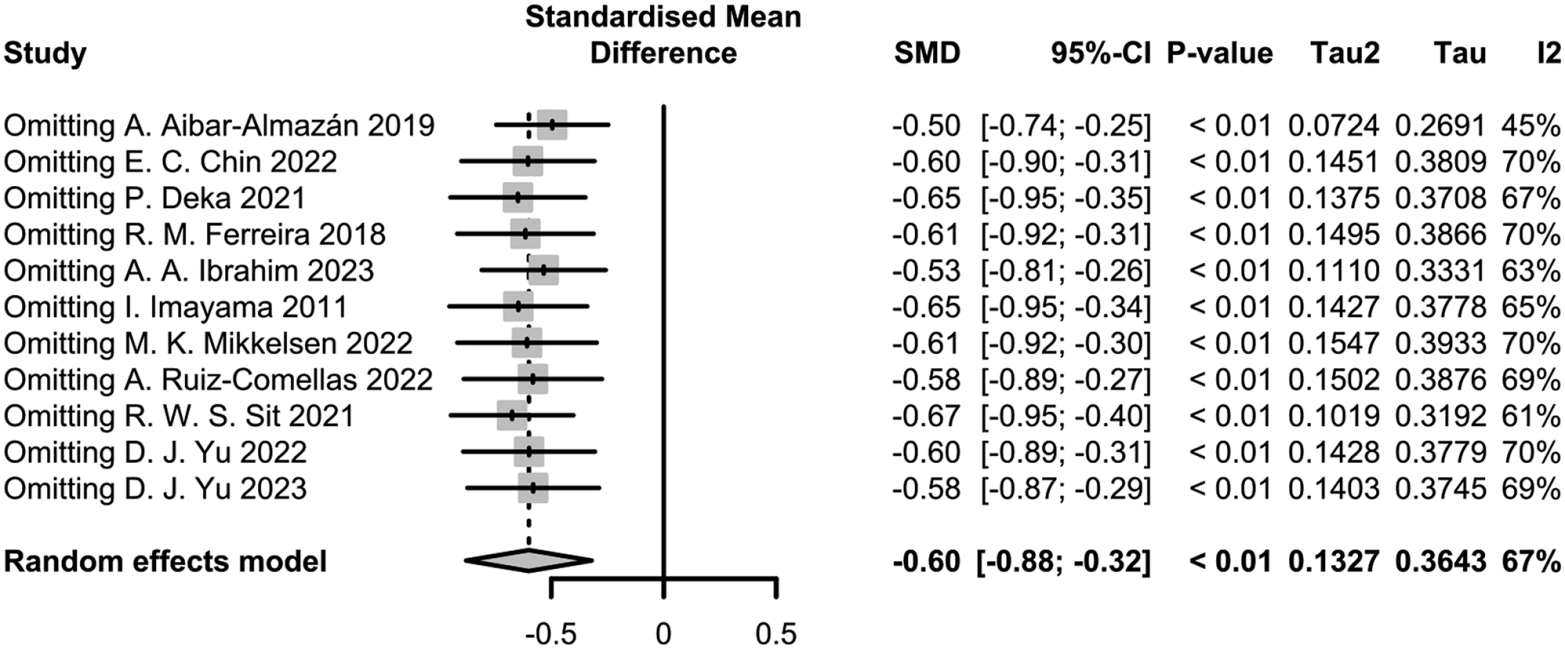
Results of sensitivity analysis

Moderate heterogeneity was observed among the included studies (I2 = 67%). Sensitivity analyses were performed by excluding one study at a time, and the overall effect size remained robust, confirming the stability of the results (Fig. 4).

### Publication bias

**Fig. 5:**
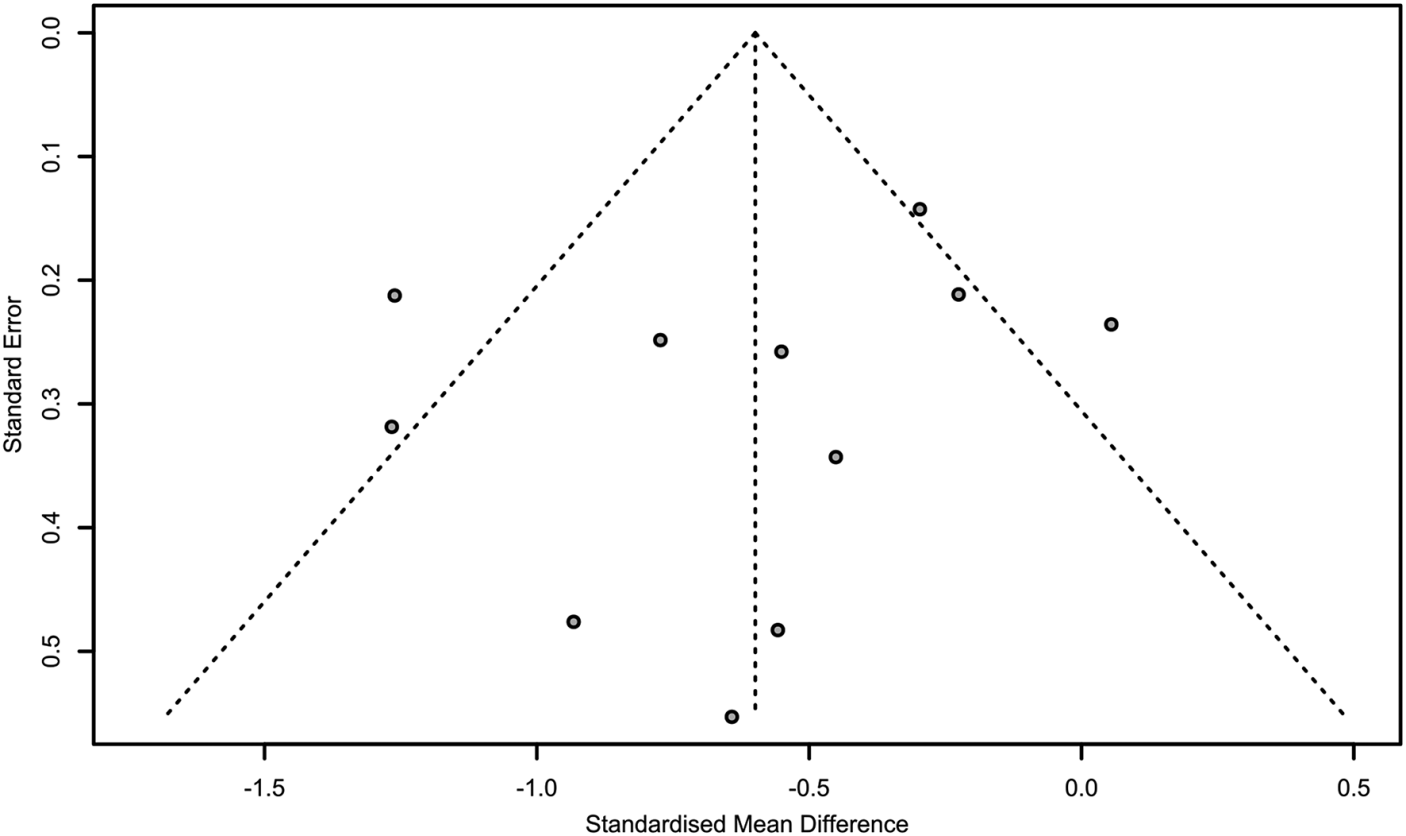
Funnel plot (Egger test: p-value = 0.36)

Visual inspection of the funnel plot suggested did not show possible signs of publication bias. Egger’s test did not reach statistical significance (*p* = 0.36), indicating no possible signs of small study effects that could impact the overall findings (Fig. 5).

### Risk of bias and quality assessment

**Fig. 6:**
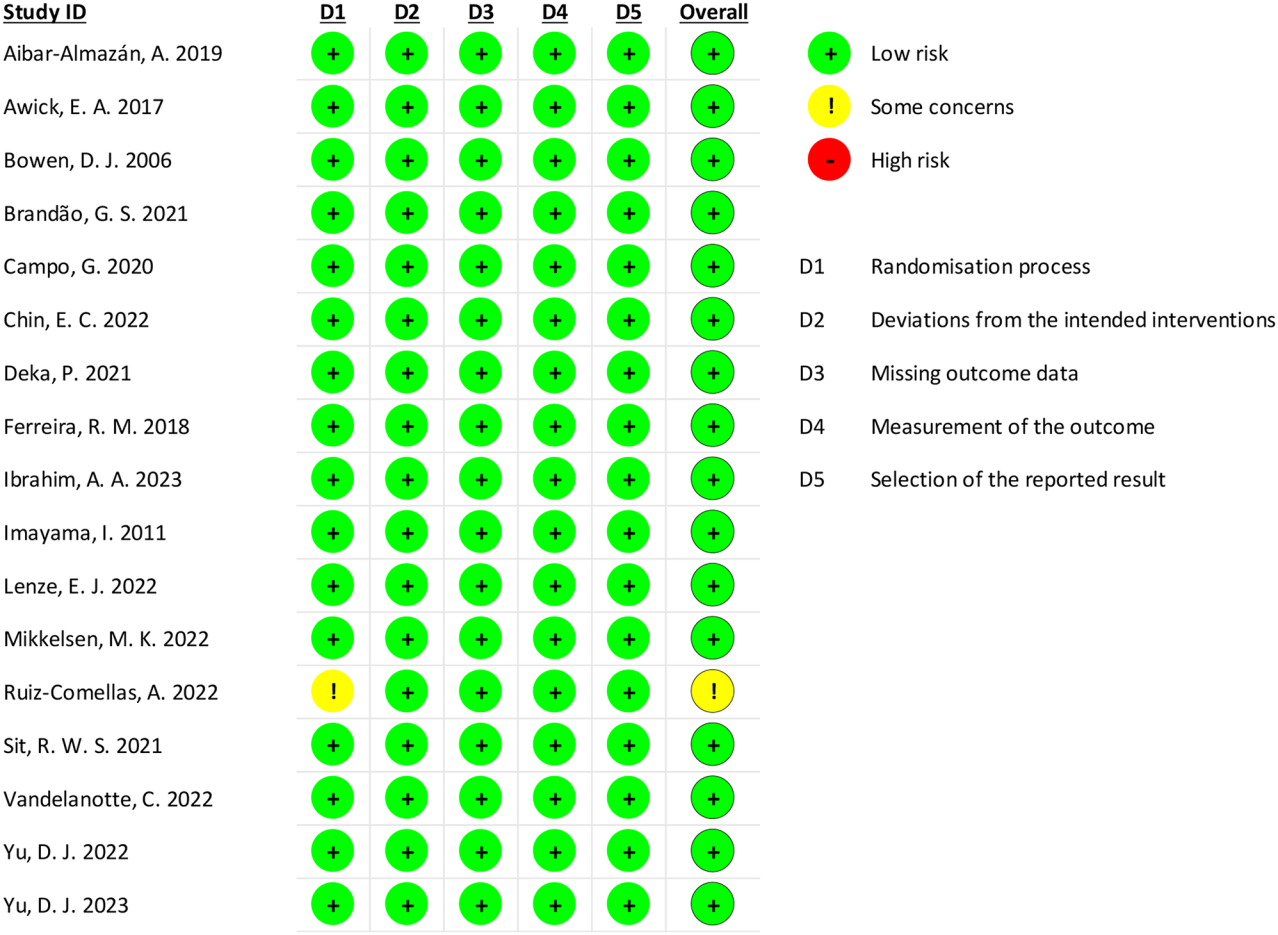
Results of risk of bias assessment

The overall risk of bias was assessed using the RoB-2 assessment tool. The overall risk of bias of included RCTs for the reduction of anxiety symptoms through physical activity was graded low, indicating a high level of confidence in the findings. (Fig. 6). The results of quality assessment are in Table 2 suggesting a moderate level of certainty regarding the primary outcome.

**Table 2:** Summary of GRADE assessment for quality of evidence

Outcome	No. of Studies	Effect Estimate	Risk Of	Inconsistency	Indirectness	Imprecision	Publication	Quality of
		(95% CI)	Bias				Bias	Evidence
								(GRADE)
Primary outcome	11	-0.60 [-0.88, -0.32]	Not serious	Not serious	Serious	Not serious	Not serious	Moderate

## Discussion

To our current understanding, this systematic review and meta-analysis represent the most exhaustive examination of the influence of physical activity on anxiety in the older adults, drawing insights from outcomes derived from peer-reviewed randomized clinical trials. Our results demonstrate a notable reduction in anxiety symptoms attributable to engagement in physical activity. Through subgroup analyses, we explored the effects of various types of physical activity. Notably, all types of exercise (including resistance trainings, cardio trainings, and combination of both) exhibited independent efficacy in alleviating anxiety. Furthermore, longer duration of exercise showed larger effect on reducing anxiety.

### The rising importance of mental health care in an aging population

The aging of global population is on the rise, emphasizing the need for increased attention to mental health care for the older adults. According to WHO, number and proportion of people aged 60 years and older in the population is increasing. By 2050, the prevalence of the world’s population over 60 years will be double from 12 to 22% [34]. This demographic shift brings attention to the importance of mental health care for older adults. Several factors, such as exposure to adversity, significant loss in intrinsic capacity, decline in functional ability, and the experience of adverse events like bereavement, retirement, and reduced income, can lead to psychological distress [35]. Older adults are more likely to experience conditions like depression and anxiety, with around 14% of adults aged 60 and over living with a mental disorder [36]. The stigma surrounding mental health conditions and the under recognition of these issues make it crucial to prioritize mental health care for the elderly [37]. Mental health problems can significantly impact an older person’s ability to carry out daily activities, emphasizing the need for early recognition, diagnosis, and treatment to prevent further decline in their well-being [38].

### Mechanisms of exercise in reducing anxiety among the elderly

Exercise has been shown to be beneficial in reducing anxiety in the elderly population through various mechanisms. Research suggests that exercise reduces anxiety by providing a time-out from daily stressors, reducing inflammation and oxidative stress, and stimulating the production of endorphins, which are the body’s natural painkillers and mood elevators [39–41]. Additionally, exercise can alleviate symptoms of anxiety by improving physical health, reducing the negative effects of stress, and improving mood. Furthermore, physiological mechanisms, such as alterations in the serotonergic and noradrenergic pathways, have been proposed as potential means through which exercise reduces anxiety [42]. The study by Ma et al. aimed to explore the factors influencing physical activity among Taiwanese adults with anxiety by testing a theoretical model using structural equation modeling. The research included 239 participants and assessed the direct and indirect impacts of 11 personal and cognitive-emotional variables on physical activity levels. The analysis led to a model that accurately fit the data, with nine of the variables explaining 23.3% of the variance in physical activity levels. The findings highlighted that perceived life stress events, perceived benefits of physical activity, and self-efficacy regarding physical activity were significant direct influencers of physical activity. These results suggest that interventions designed to enhance physical activity in Taiwanese adults with anxiety should specifically address these factors to be more effective [43]. Therefore, the evidence supports the use of exercise as an effective intervention for reducing anxiety in the elderly population, with multiple biological and psychological mechanisms contributing to its beneficial effects.

### Role of different type of exercises in reducing anxiety

Numerous studies have shown that aerobic exercise, such as walking, running, and cycling, can significantly reduce anxiety symptoms in older adults. The physiological mechanisms include the release of endorphins and improved cardiovascular health, which collectively enhance mood and reduce stress levels [44]. For instance, a meta-analysis by Yao et al. demonstrated that older adults engaging in aerobic exercises experienced a significant reduction in anxiety [45]. Resistance training, involving activities such as weightlifting and resistance band exercises, has also been found to alleviate anxiety symptoms [46]. This form of exercise not only improves muscle strength and endurance but also contributes to psychological well-being. A mixed method systematic review by Li et al. [47] reported that older participants who engaged in resistance elastic band training showed significant improvements in their mental health. As showed in our study, engaging in group-based exercises, such as group walking or group dance classes, can also provide social support and a sense of community, which are crucial for mitigating anxiety in older adults [48]. Social interaction during these activities can reduce feelings of isolation and loneliness, further contributing to decreased anxiety levels [49].

As we found in the results of our subgroup analysis, both cardio and resistance exercise have benefits for reducing anxiety levels in older adults. However, no unique RCT or network meta-analysis have been performed to compare the efficacy of each type of aforementioned exercises (or a combination of both) on anxiety levels among geriatrics. Future studies are warranted to yield this gap of knowledge.

### Implications

In terms of clinical implication, the research on the effect of exercise on anxiety in the elderly is important as it provides evidence that physical activity is among the most important, simplest, and cheapest approaches to anxiety treatment for this population [50]. This research can help improve the care given to the elderly population with anxiety by highlighting the effectiveness of exercise in reducing anxiety symptoms, providing a non-invasive and accessible intervention, and emphasizing the importance of early recognition and appropriate treatment to prevent negative consequences associated with anxiety in older adults. Additionally, understanding the mechanisms through which exercise reduces anxiety can inform the development of tailored exercise interventions to better support the mental health needs of the elderly population, improving public health.

### Strengths and limitations

Our investigation delves into a topic that has been the focus of two other systematic review and meta-analysis studies [21, 51]. However, our study stands out for its methodological robustness, boosted by several advantages such as: (1) selective inclusion of Randomized Controlled Trials (RCTs), renowned for their stringent methodologies and controlled environments. This strict approach contributes to the heightened reliability of outcomes when compared to studies that include studies with different research types; (2) our analyses integrate more recent studies with updated methodologies and results, reinforcing the relevance of our findings; (3) the quality of evidence of the studies included in our analyses was assessed as high; (4) we conducted subgroup analyses to systematically evaluate the impact of potential confounding factors, such as variations exercise, and duration; and 4) the heterogeneity of results among the studies included in our investigation is noticeably lower than the mentioned studies. It is important to acknowledge the limitations we encountered. First, the included studies had relatively small participant numbers, prompting the need for future research with larger groups to enhance the certainty and accuracy of our results. This consideration is pivotal for advancing our comprehension of the subject matter. Second, there was no study with main focus on comparing different types of exercises together. Finally, few studies have been conducted on the effect of resistance exercises. The majority of included studies were focused on cardio exercises or a combination of both cardio and strength training.

## Conclusion

This systematic review and meta-analysis provide compelling evidence of RCTs on the significant impact of physical activity on reducing anxiety symptoms in the elderly population. The demonstrated efficacy of physical activity, particularly cardio and strength exercises, in alleviating anxiety, underscores the importance of incorporating tailored exercise interventions into strategies for anxiety management in the elderly. The limitations related to the relatively small participant numbers in the included studies highlight the need for future research with larger groups to enhance the certainty and accuracy of our results.

## Data Availability

All data has been presented in the manuscript.
